# Lung enteric-type adenocarcinoma with gastric metastasis: a rare case report and literature review

**DOI:** 10.3389/fimmu.2024.1486214

**Published:** 2024-10-23

**Authors:** Xiaoning Li, Kewei Ma, Xiaobo Ma, Xiangye Zhao, Mengge Fan, Yinghui Xu

**Affiliations:** ^1^ Cancer Center, The First Hospital of Jilin University, Changchun, Jilin, China; ^2^ Department of Pathology, The First Hospital of Jilin University, Changchun, Jilin, China

**Keywords:** lung enteric-type adenocarcinoma, gastric metastasis, NRAS gene exon 3 mutation, chemotherapy and immunotherapy, non-small cell lung cancer

## Abstract

Lung enteric-type adenocarcinoma (ETAC) is a rare subtype of non-small cell lung cancer (NSCLC), comprising approximately 0.6% of all primary lung adenocarcinomas. It is characterized by a tendency for early metastasis and a prognosis comparable to that of common lung adenocarcinoma. This case report described a patient with lung-ETAC who developed gastric metastasis. The patient underwent treatment with chemotherapy and a PD-1 inhibitor, resulting in disease remission with a progression-free survival (PFS) of 8 months. The follow-up time was 13 months. This case report was aimed to enhance understanding of the biological behavior of this rare tumor and provide insights into potential future treatment strategies.

## 1 Introduction

Lung enteric-type adenocarcinoma (ETAC) is a rare and distinct subtype of non-small cell lung cancer (NSCLC), accounting for approximately 0.6% of all primary lung adenocarcinomas. It was firstly described by Tsao and Fraser in 1991 (1). It originates in the lungs but exhibits intestinal differentiation in its pathology. In 2015, this subtype was officially recognized in the World Health Organization (WHO) classification of lung tumors (2). The 2021 WHO classification redefined this entity as lung-ETAC. Histological diagnostic criteria require more than 50% enteric morphology (3). Due to its histological and immunohistochemical similarities to metastatic colorectal cancer (mCRC) (4), differential diagnosis often necessitates endoscopy, computed tomography (CT), and especially 18F-labeled fluoro-2-deoxyglucose positron emission tomography (18F-FDG PET-CT) to rule out primary gastrointestinal tumors (5, 6) It is an aggressive tumor with a propensity for early metastasis, often spreading to bones, liver, and lymph nodes (7), and occasionally affecting the skin, scalp, brain, pancreas, and soleus muscle (8–10). However, gastric metastasis has not been previously reported. This report described a case of lung-ETAC with gastric metastasis.

## 2 Case report

**Figure 1:**
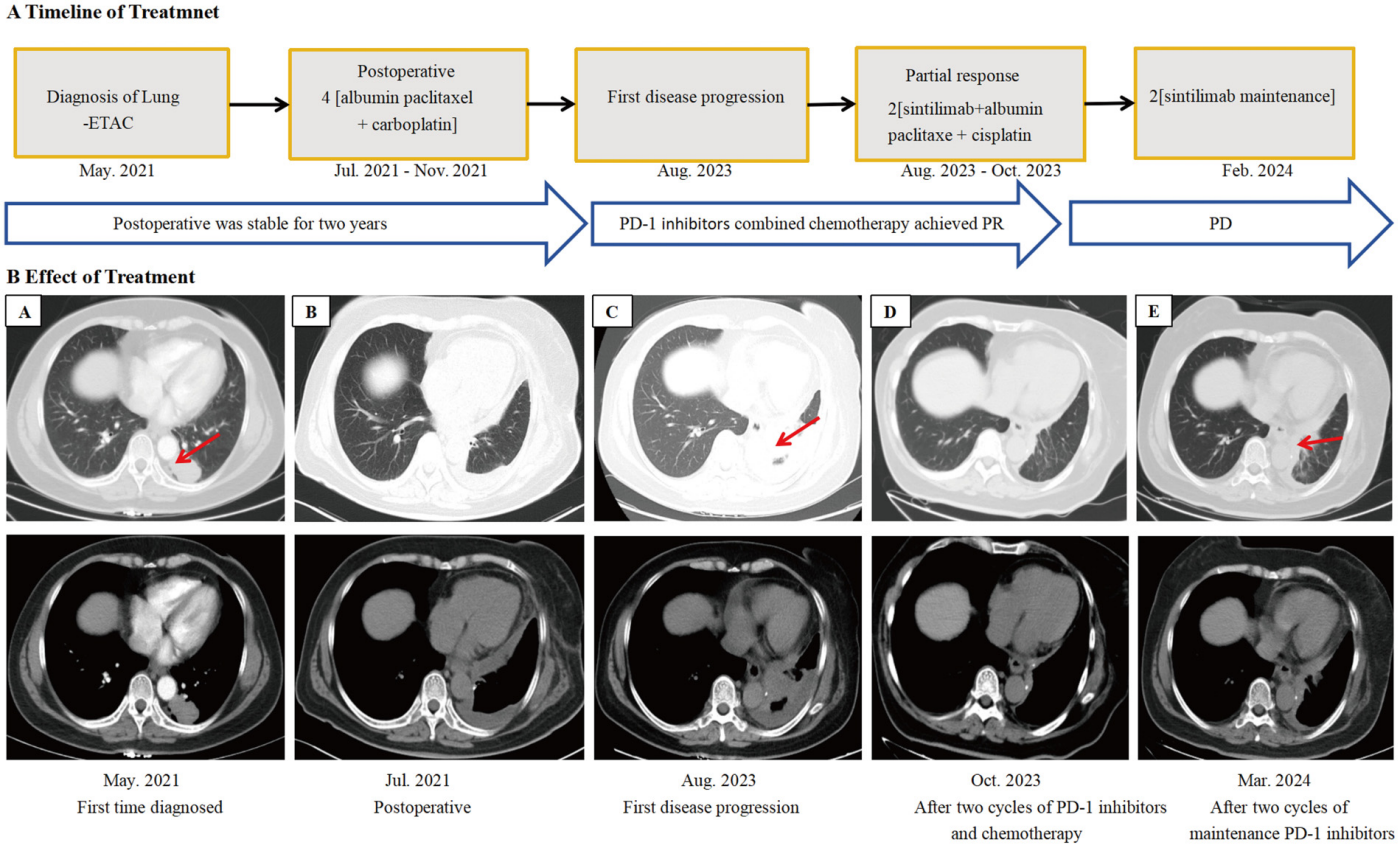
Imaging changes during treatment. CT images demonstrating diagnosis, treatment responses, and progression. **(A)** Lung-ETAC was first diagnosed (May 2021), enhanced CT scan showed a 2.6x3.8cm pulmonary nodule of the left lung. **(B)** Postoperative (Jul 2021). **(C)** Progression after postoperative, chest CT scan showed high-density shadows of bronchia in the left lung and larger than before (Aug 2023). **(D)** Partial remission was achieved after two courses of PD-1 inhibitors in combination with chemotherapy (Oct 2023). **(E)** Progression after two courses of maintenance immunotherapy (Mar 2024). PR, partial response; PD, disease progression.

**Figure 2:**
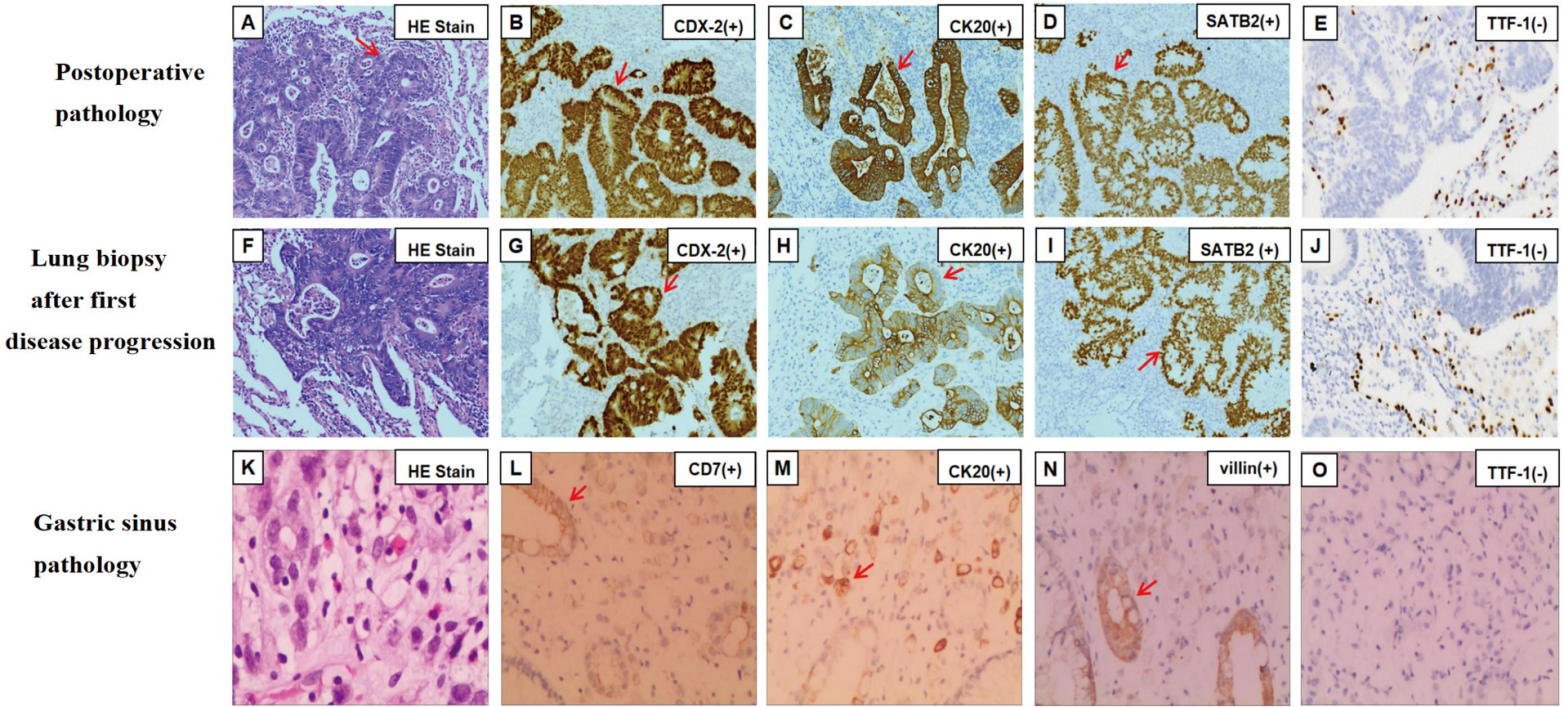
Hematoxylin and eosin staining and immunohistochemical findings of samples at different disease courses. **(A)** Postoperative pathology of left lung on hematoxylin and eosin staining. **(B-E)** Immunohistochemical staining was positive for CDX-2 **(B)**, CK20 **(C)**, and SATB2 **(D)**. **(F)** Hematoxylin and eosin staining of lung biopsy after first disease progression. **(G-J)** Immunohistochemical staining was positive for CDX-2 **(G)**, CK20 **(H)**, and SATB2 **(I)**. **(K)** Gastric sinus on hematoxylin and eosin staining. **(L-O)** Immunohistochemical staining was positive for CK7 **(L)**, CK20 **(M)**, villin **(N)**. Magnification×200 **(A-O)**. The red arrow points out positively stained cells. TTF-1, thyroid transcription factor-1; CK, cytokeratin; CDX-2, caudal-type homeobox transcription factor 2; SATB2, Special AT-Rich Sequence-Binding Protein 2.

A 63-year-old female was admitted to the hospital in May 2021 for physical examination, which revealed a nodule in the left lower lobe of the lung. Chest CT demonstrated that the nodule was located in the lower lobe of the left lung, measuring 2.6x3.8cm (**Figure 1A**). Bone scan and brain CT results were normal. On May 28, 2021, the patient underwent a left-sided thoracoscopic lower lobe resection plus lymph node dissection (**Figure 1B**). Postoperative pathology confirmed invasive adenocarcinoma, enteric type. Immunohistochemistry (IHC) results were as follows: CKpan (+), CK7 (partially +), CK20 (+), CDX-2 (+), Ki-67 (+80%), TTF-1 (-), NapsinA (-), SATB2 (+), Villin (+), CK5/6 (-) (**Figures 2B-E**). The diagnosis was lung-ETAC (T2aN1M0 IIb). The patient received four courses of adjuvant chemotherapy with albumin paclitaxel and carboplatin, followed by long-term follow-up. In July 2023, the patient presented with symptoms of choking sensation on food and chest pain, and disease progression was noted. The disease-free survival (DFS) was 26 months. Chest CT revealed high-density shadows in the left lung bronchus. (**Figure 1C**). PET-CT confirmed these findings and identified bone and gastric sinus metastasis. Fiberoptic bronchoscopy and biopsy were performed, and the pathology of the left lung bronchus confirmed adenocarcinoma, enteric type, with PD-L1 expression at 20%. IHC results were: CDX-2 (+),SATB2 (+), CK20 (+), TTF-1 (-) (**Figures 2G-J**). Gastroscopy on August 28, 2023, revealed an erosion in the gastric antrum and the lesser curvature of the stomach. Pathological examination of the gastric sinuses showed poorly differentiated carcinoma. IHC results were: CK (+), CK7 (scattered +), CK20 (+), CEA (+), villin (weakly +), P53 (-), Ki-67 (+ about 20%), Syn (-), CD56 (-), CD45 (-), E-Cad (+), Her-2 (-), TTF-1 (-), Napsin-A (-) (**Figures 2L-O**). Genetic testing of the left lung bronchus identified an NRAS gene exon 3 mutation. Histopathological comparison by an experienced specialist revealed that the gastric and bronchial pathologies contained moderately and poorly differentiated adenocarcinomatous components similar to the postoperative lung pathology, confirming gastric metastasis. The patient was ultimately diagnosed with left lung-ETAC (cT4N3M1, stage IV) with lung, bone, and gastric sinus metastasis. The patient received six cycles of sintilimab (anti-PD1, 200mg) - albumin paclitaxe (260mg/m2), - and cisplatin (75mg/m2), the patient got a partial response after two cycles (**Figure 1D**), then with two courses of sintilimab (anti-PD1) immunotherapy maintenance. In March 2024, the therapeutic assessment suggested disease progression (**Figure 1E**) and PFS was 8 months. The patient refused further treatment. Finally, The patient received the best supportive care. At this writing, the patient remains alive but in generally poor condition. The follow-up time was 13 months.

## 3 Discussion

Our report described a case of primary lung-ETAC with gastric sinus metastasis, accompanied by an NRAS gene exon 3 mutation. After relapse, the patient underwent treatment with a combination of PD-1 inhibitors and chemotherapy. Six courses of this combination were administered, followed by two cycles of maintenance immunotherapy. Partial remission was achieved after two courses of PD-1 inhibitors in combination with chemotherapy, but progression was observed after two cycles of immunotherapy maintenance, as indicated by imaging assessments. The PFS was 8 months.

Among the rare subtypes of NSCLC, including enteric, fetal, and colloid types, the enteric type represented approximately 60% of these cases (11). The etiology of lung-ETAC remains unclear, with hypotheses suggesting common cancer stem cells in the mucosa of the lower respiratory and gastrointestinal tracts and the activation of the Wnt/β-catenin signaling pathway (12, 13). There was also an association found between lung-ETAC and smoking (14). Studies have shown that lung-ETAC is more prevalent in males, typically found in the right lung, and is rare in the left lung (15). In our case, the patient had no history of smoking, and the primary lesion was located in the left lung.

Histological features of lung-ETAC include well or moderately-differentiated gland formation and/or papillary structures. Tumor cells are usually tall-columnar with slightly darker-stained nuclei and nuclear palisading (16) (**Figures 2A, F, K**), similar to CRC. Previous studies have confirmed that CK20, CDX-2, SATB2, villin, and HNF4α are common biomarkers of digestive tract epithelium, and TTF-1 and NapsinA are biomarkers of the alveolar epithelium (17, 18). The diagnosis of lung-ETAC is often supported by IHC. Lung-ETAC expresses at least one enteric biomarker, such as CDX-2, CK20, villin, MUC2, HNF4α, and SATB2, while lung cellular markers like TTF-1 and NapsinA are generally absent. CK7 is usually expressed in contrast to lung adenocarcinoma (19). This was consistent with our case, where IHC analysis of the lung tissue revealed positive results for CDX-2, CK20, and villin, a partial positive result for CK7, and negative staining for TTF-1 and NapsinA. No gastrointestinal lesions were detected at the initial diagnosis, only lung lesions. KRAS, NRAS, and EGFR are common oncogenes in lung-ETAC. It is reported that the incidence of KRAS and NRAS mutations was about 31% (15, 20–22). KRAS is a genetic profile of invasive mucinous adenocarcinoma of the lung (23). In this case, the NRAS mutation was predominantly identified, the most common gene mutations in lung cancer are all negative, including EGFR, KRAS, BRAF, ALK, ROS1, and so on. Within rare oncogenes of NSCLC, NRAS mutations occur in only 0.9%, with a high frequency of codon 61 mutations (24). Mutations in KRAS/NRAS (RAS) are important biomarkers in mCRC and occur in approximately 60% of mCRCs (25). Mutant RAS proteins exert oncogenic effects through signaling pathways, with tumor cells carrying mutant RAS exhibiting a more aggressive phenotype. RAS-RAF-MEK-ERK and RAS-PI3K-AKT-mTORC are the basic signaling pathways of RAS proteins (26). The mutational status of RAS correlates with a worse prognosis. NRAS also promotes the colonization of the lungs by various tumor types in mouse models by regulating interleukin-8-related chemokine expression (27). Direct targeting of RAS proteins was previously considered impossible due to the lack of binding pockets on the protein surface; however, the approval of Lumakras (sotorasib, AMG510) for KRAS mutations has changed this perception (26). Currently, there are no targeted agents for NRAS mutations, so we opted for a combination of chemotherapy and immunotherapy based on positive PD-L1 expression.

Common extrapulmonary metastatic sites for lung cancer include lymph nodes, the brain, liver, adrenal glands, and bones. Gastric metastasis from lung cancer has been relatively rare, with incidence rates ranging from 0.19% to 5.1% (28). However, the autopsy detection rate is notably high (29, 30). The most frequently affected gastric sites are the fundus and cardia (29). It is hypothesized that gastric metastasis may be related to the stomach’s rich vascular system, which facilitates hematogenous dissemination (31). In this case, the diagnosis of lung ETAC with gastric metastasis was established following PET-CT and the integration of pathological features from both the lung and stomach. To date, gastric metastasis from lung-ETAC has not been reported. The NRAS mutation was predominantly identified in our report. Compared to lung cancer, NRAS mutations are more common in CRC, the percentage approximately 60% of mCRC, and are associated with strong aggressiveness (25, 26). It has been reported in the literature that KRAS mutations were associated with CRC metastasis (32), NRAS promotes the colonization of the lungs by various tumor types in mouse models (27). Whereas whether NRAS mutations mediate gastric metastasis needs to be further confirmed. Due to the specificity of the pathologic type, its morphological and molecular level alterations preserved features of CRC. So we speculate that this rare subtype may have a propensity to metastasize to the gastrointestinal tract.

The prognosis of lung-ETAC is not well understood due to its low incidence, and it is closely related to its clinical stage. Gu et al. reported that survival times for stage III or IV patients ranged from 0 to 9 months, whereas stage I or II patients had survival times ranging from 1 to 27 months. Fassi et al. found that the median overall survival (mOS) was 56.0 months for early-stage patients and 14.0 months for those with advanced or metastatic disease, with a median DFS (mDFS) of 24 months. This was consistent with our case. Wang et al. reported that PFS for 15 patients with stage III-IV ranged from 2.0 to 17 months, with a mean PFS of 6.5 months and a median PFS (mPFS) of 6.0 months (15, 33, 34). Several factors have been identified as potentially influencing prognosis, including smoking history, Eastern Cooperative Oncology Group (ECOG) performance status, tumor size ≤ 5 cm, lymph node metastasis, and expressions of biomarkers such as CK20, CDX-2, and NapsinA (15, 34–37). Chemotherapy remains the predominant treatment approach and can achieve complete remission or stability in some cases (38–40). Lung-ETAC appears to benefit from immunotherapy as well. Chen et al. were the first to identify that the nonsynonymous tumor mutational burden (TMB) of lung-ETAC was significantly higher compared to typical pulmonary adenocarcinomas (20). Jurmeister et al. first reported positive membrane PD-L1 staining in lung-ETAC tumor cells (41). Manglaviti et al. found the efficacy of immune-checkpoint inhibitors (ICIs) remains uncertain in uncommon histologies (35). Xie et al. and Liu et al. observed the PD-L1 expression in 17.6% (3/17) and 30% (3/10) of patients, respectively (22, 42). Despite these observations, the efficacy of immunotherapy is still debated. Gastrointestinal tumor regimens such as FOLFOX have also been used due to the pathology’s specificity (15). Targeted therapies have been promising with one case of EGFR L858R + A871G mutations treated with gefitinib achieving 5 years of PFS, and another case with EGFR E19del and T790M mutations treated with osimertinib showing 3 months of PFS (43, 44). We compared similar studies, and the treatment regimens are summarized in **Supplementary Table 1** (48–56). Most of the studies were treated with conventional chemotherapy with a PFS of 3-5 months. In our case, PD-L1 expression is 20%, which is moderate. In NSCLC, gastric cancer, and gastroesophageal junction tumors, PD-L1 expression might be useful as a predictive marker of response to anti-PD1 therapy (45). So the patient was treated with a combination of PD-1 inhibitor and chemotherapy after gastric metastasis, resulting in a PFS of 8 months. This confirms that PD-1 inhibitors in combination with chemotherapy are significantly better than chemotherapy. This was consistent with findings from the KEYNOTE-189, IMpower150, and ORIENT-11 studies, which showed that immunotherapy combined with chemotherapy significantly improved PFS in previously untreated patients with metastatic non-squamous NSCLC, with PFS rates of 9, 6.3, and 11.4 months, respectively (46, 47). We reviewed the literature and were unable to conclude that PD-1 inhibitors prevented gastric metastasis.

Currently, data on the treatment and prognosis of lung-ETAC have been limited, with most information derived from retrospective studies. This case highlighted the need for enhanced pathological and molecular diagnostics and calls for more comprehensive studies to better understand the clinical benefits and prognosis of lung-ETAC.

In conclusion, this study reported the first case of lung-ETAC with gastric metastasis, demonstrating the effectiveness of chemotherapy in combination with immunotherapy. However, the mechanisms of gastric metastasis need further clarification.

## Data Availability

The original contributions presented in the study are included in the article/**Supplementary Material**. Further inquiries can be directed to the corresponding author.
