# Supplementary material for: Lung enteric-type adenocarcinoma with gastric metastasis: a rare case report and literature review
Source: Front Immunol. 2024 Oct 23;15:1486214. doi: 10.3389/fimmu.2024.1486214 (PMC11537902; doi:10.3389/fimmu.2024.1486214)
Supplement: Supplementary file 1 [file Table1.docx]

Supplementary Material

# Supplementary Tables

We summarized the therapy of each case report/review in **Table 1**.

| **Author/Year** | **Type of Study** | **Number of patients** | **PD-L1/TMB** | **Molecular data** | **Disease Stage** | **Neoadjuvant therapy** | **Surgery** | **Adjuvant therapy** | **First-line** | **PFS**  **(months)** | **Second-line** | **PFS (months)** | **Other therapy** | **OS (months)** | **Reference** |
| --- | --- | --- | --- | --- | --- | --- | --- | --- | --- | --- | --- | --- | --- | --- | --- |
| Li et al./2009 | Case report/Review | 1 | - | - | IIB | NO | Yes, DFS=10months | 4 cycles of Chemotherapy | 6 cycles of pemetrexed + carboplatin, SD | - | NO |  |  | - | (40) |
| Qureshi et al./2013 | Case report | 1 | - | EGFR | IV | NO | Yes | NO | 4 cycles of pemetrexed + cisplatin, PR | - | NO |  |  | - | (38) |
| Metro et al./2015 | Case report | 1 | - | KRAS Q22K | IV |  | NO |  | 3 cycles of single-agent gemcitabine | 5 | 2 cycles of pemetrexed | 1.5 | NO | about 7 | (48) |
| Garajová et al./2015 | Case report | 2 | - | KRAS | IIB | NO | Yes |  | 2 cycles of XELOX (capecitabine + oxaliplatin), PD | 4 | 4 cycles of carboplatin + pemetrexed, PD | 3 | 2 cycles of docetaxel, SD | - | (49) |
|  |  |  | - | - | IB | NO | Yes，DFS=12months | NO | 6 cycles of carboplatin + pemetrexed, pemetrexed maintenance, SD | 5 | NO |  |  | - |  |
| Lin et al./2016 | Case report/Review | 1 | - | EGFR wild-type、 KRAS、BRAF、UGT1A1 | IV |  | NO |  | 3 cycles of XELOX chemotherapy regimen, PD | 3 | 4 cycles of TP regimen, 2 cycles of FOLFIRI regimen, | 6 | DP, SD | - | (50) |
| Lin et al./2017 | Case series | 11 | - | ALK/ROS1（5/11）、BRCA2（1/11）、BRAF （1/11）、NRAS （1/11）PIK3CA（1/11）、MSH2/MSH6（3/11） | IIIB-IV | NO | 1:Yes  1：neoadjuvant chemotherapy，then surgery | NO | 1: icotinib (1.5 months) + nivolumab (9.5 months) others: chemotherapy | - |  |  |  | mOS：9.0 months，LAK/ROS1: mOS：6.5 months，MSH2/MSH6: mOS: 26 months | (51) |
| Prakobkit et al./2017 | Case report | 1 | - | - | IV |  |  |  | 1 cycles of carboplatin + paclitaxel | - |  |  |  | - | (52) |
| Todisco et al./2019 | Case report | 1 | - | KRAS、CDKN2A | IV |  | NO | - | 2 cycles of pemetrexed + cisplatin | 1.5 |  |  |  | 1.5 | (8) |
| Tu et al./2021 | Case report | 6 | TMB (low or medium) | KRAS (2/4) | IA-II: 2pts | NO | 1: Yes | NO |  |  |  |  |  | 1:58 month，  1:7 months  1:9 month,  3pts lost to follow-up | (53) |
|  |  |  |  |  | IV: 4pts |  |  |  | 1: 4 cycles of pemetrexed + cisplatin, PD 1: TC+ bevacizumab, SD 1: Pemetrexed + Carboplatin, SD | - |  |  |  |  |  |
| Teranishi et al./2022 | Case report | 1 | PD-L1 TPS＜1% | KRAS G12D | IVB |  | NO | - | 4 cycles of pembrolizumab+carboplatin+pemetrexed,3 cycles of pembrolizumab+ pemetrexed, SD | ＞10 |  |  |  | - | (54) |
| Xu et al./2022 | Case report/Review | 1 | PD-L1 TPS 0% | EGFR exon 19、KRAS G12V、TP53 | IIA |  | NO | - | 4 cycles of pemetrexed + cisplatin+ penpulimab, SD | - |  |  |  | - | (38) |
| Hu et al./2022 | Case report | 1 | - | KRAS G12C | IVB |  | NO | - | 1 cycles of paclitaxel  +carboplatin+ sindilizumab, PD | 1 |  |  |  | - | (55) |
| Cui et al./2023 | Case report | 1 | - | EGFR L858R + A871G、TP53、 | IIIB | NO | Yes | 1 cycles of pemetrexed +cisplatin | Gefitinib | ＞62 |  |  |  | - | (43) |
| Fassi et al./2023 | Case report/Review | 1 | PD-L1＜1% | - | IV |  | NO | - | 6 cycles of FOLFOX (Oxaliplatin+ leucovorin + fluorouracil), PR fluorouracil | 15 | FOLFIRI | 8 | NivolumabPFS=9 months | 34 | (15) |
| Yang et al./2024 | Case report | 1 | TMB-H：29.76Muts/Mb | EGFR E19del、T790M、MSI-H、MMR | IV |  | NO | - | Icotinib | 17 | Osimertinib | 3 | 2 cycles of nedaplatin+ pemetrexed+bevacizumab,4 cycles of nedaplatin+pemetrexed,1 cycles of pemetrexed PFS=6.5months camrelizumab±pemetrexed,gefitinib | 30 | (44) |
| Nemoto et al./2024 | Case report | 1 | - | No | IIIA | 5 cycles of weekly carboplatin + paclitaxel (CBDCA+ PTX) + 66 Gy/33 Fr | Yes, DFS=3months，then metastatic brain tumor performed surgery and 45 Gy/15 Fr | Chemothreapy | NO |  |  |  |  | ＞28 | (56) |

**
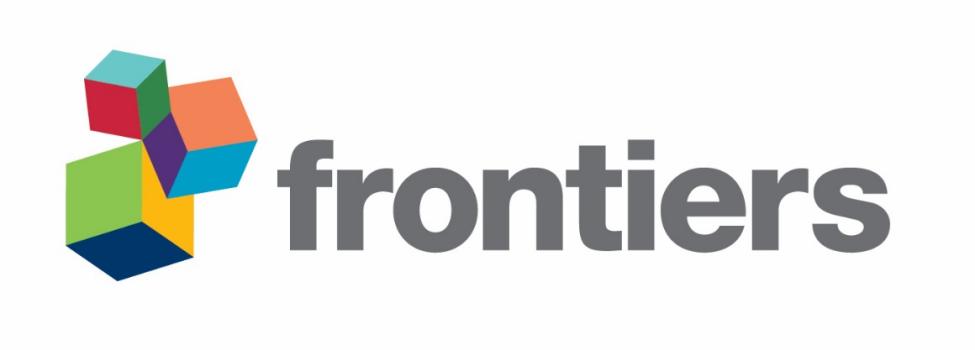
**
